# Circular RNA circRHOT1 promotes hepatocellular carcinoma progression by initiation of *NR2F6* expression

**DOI:** 10.1186/s12943-019-1046-7

**Published:** 2019-07-19

**Authors:** Liyan Wang, Haiyan Long, Qinghua Zheng, Xiaotong Bo, Xuhua Xiao, Bin Li

**Affiliations:** Gastrointestinal Department, Affiliated Hospital of Guilin Medical University, Lequn road No.15, Xiufeng district, Guilin, 541001 China

**Keywords:** circRHOT1, Hepatocellular carcinoma, TIP60, NR2F6

## Abstract

**Background:**

Increasing evidence has revealed a close relationship between non-coding RNAs and cancer progression. Circular RNAs (circRNAs), a recently identified new member of non-coding RNAs, are demonstrated to participate in diverse biological processes, such as development, homeostatic maintenance and pathological responses. The functions of circRNAs in cancer have drawn wide attention recently. Until now, the expression patterns and roles of circRNAs in hepatocellular carcinoma (HCC) have remained largely unknown.

**Methods:**

Bioinformatics method was used to screen differentially expressed novel circRNAs in HCC. Northern blotting, qRT-PCR, in situ hybridization (ISH) and RNA-FISH were utilized to analyzed the expression of circRHOT1 in HCC tisues.CCK8, colony formation, EdU assays were used to analyze proliferation of HCC cells. Transwell assay was utilized to analyze HCC cell migration and invasion. FACS was used for apoptosis analysis. Xenograft experiments were used to analyze tumor growth in vivo. Mass spectrum, RNA pulldown, RIP and EMSA was utilized to test the interaction between circRHOT1 and TIP60. RNA-sequencing method was used to analyze the downstream target gene of circRHOT1.

**Results:**

We identified circRHOT1 (hsa_circRNA_102034) as a conserved and dramatically upregulated circRNA in HCC tissues. HCC patients displaying high circRHOT1 level possessed poor prognosis. Through in vitro and in vivo experiments, we demonstrated circRHOT1 significantly promoted HCC growth and metastasis. Regarding the mechanism, we conducted a RNA pulldown with a biotin-labeled circRHOT1-specific probe and found that circRHOT1 recruited TIP60 to the *NR2F6* promoter and initiated *NR2F6* transcription. Moreover, NR2F6 knockout inhibited growth, migration and invasion, whereas rescuing NR2F6 in circRHOT1-knockout HCC cells rescued the proliferation and metastasis abilities of HCC cells.

**Conclusion:**

Taken together, circRHOT1 inhibits HCC development and progression via recruiting TIP60 to initiate *NR2F6* expression, indicating that circRHOT1 and NR2F6 may be potential biomarkers for HCC prognosis.

**Electronic supplementary material:**

The online version of this article (10.1186/s12943-019-1046-7) contains supplementary material, which is available to authorized users.

## Background

Currently, hepatocellular carcinoma (HCC) has become a most prevalent cancer worldwide [1]. This malignancy contributes to a great proportion of cancer-related death each year, especially in China [2]. Until now, the main therapeutic methods for HCC have included curative resection and chemotherapy. In past decades, efforts and advances have been made concerning HCC treatment. However, the prognosis of HCC patients is still very poor, and the 5-year survival rate is lower than 25% due to tumor recurrence and metastasis with a high frequency [3]. Therefore, these serious challenges make it very urgent to find novel biomarkers for HCC diagnosis and identify effective therapeutic targets.

Recently, circular RNAs (circRNAs) have attracted much attention. CircRNAs are a new member of non-coding RNAs that have little protein coding potential and make up more than 90% of the transcriptome in humans [4, 5]. Circular RNAs are derived from precursor mRNA back splicing and are covalently closed transcripts [6]. Therefore, circRNAs are more stable and resistant to decay machineries than linear RNAs [7]. Some studies have shown that the formation of circRNAs relies on the presence of flanked complementary sequences such as Alu elements [8]. High-throughput sequencing has reveal that the expression of circRNAs is cell, tissue or development specific, suggesting that circRNAs may have specific biological functions [9]. Increasing evidence has shown that circRNAs are potential mediators in tumor biology via diverse mechanisms [10]. For instance, ciRS-7 works as a sponge for miR-7 in brain [11]. circ-Foxo3 induces tumor cell apoptosis by enhancing Foxo3 activity [12]. In HCC, some studies also revealed the existence of numerous circRNAs, such as circMTO1 and CircARSP91 [7, 10]. However, the function and mechanism of circRNA in HCC need to be further explored.

In our study, we analyzed two circRNA profiles expressed in human HCC tissues and identified circRHOT1 (hsa_circRNA_102034) as a conserved and significantly upregulated circRNA in HCC tissues. Additionally, the expression of circRHOT1 was closely related to the HCC patient prognosis. We found that circRHOT1 could recruit TIP60 to the *NR2F6* promoter, initiate *NR2F6* expression and eventually promote HCC progression. Thus, circRHOT1 and NR2F6 might act as novel biomarkers for HCC prognosis and promising therapeutic targets.

## Methods

### HCC samples

We obtained 100 paired HCC tissues and adjacent normal tissues from the Affiliated Hospital of Guilin Medical University. The relationship between circRHOT1 expression and clinical severity is listed in Additional file 1: Table S1. Samples receiving chemotherapy or radiotherapy before collection were excluded. Written consent approving the usage of HCC tissues in our study were obtained from each patient. This study was approved by the ethnic committee of the Affiliated Hospital of Guilin Medical University.

### Cell culture

For primary HCC cell culture, the surgical-isolated HCC tissues were digested with collagenase IV and hyaluronidase for 50 min at 37 °C. Afterwards, the cell suspensions were washed, filtered using a 70 μm cell strainer (BD, USA) and seeded on collagen-coated Petri dishes. Fibroblasts and blood vessels were discarded. The primary cancer cells were cultured in DMEM medium supplemented with 20% FBS, 2 mM glutamine, 1 mM pyruvate, 10 mM HEPES, 100 units/mL penicillin/streptomycin, 0.1 mg/mL gentamicin, and 2 g/L Fungizone as previously reported [13]. The primary cells at passage 4~10 were used for experiments.

### CRISPR-Cas9 knockout system

circRHOT1, TIP60 or NR2F6 knockout and NR2F6 promoter deletion in HCC cells were established using the CRISPR/Cas9 system as previously described [14, 15]. Briefly, we designed single guide RNAs (sgRNA) using an online tool (http://crispr.mit.edu/) and cloned them into the lenti-Cas9-eGFP vector (Addgene, 63592). Next, 293 T cells were transduced with the lenti-Cas9-eGFP-sgRNA as well as pVSVg (Addgene, 8454), and psPAX2 (Addgene, 12260) to produce lentivirus. The viruses were concentrated with PEG5000 (Sigma Aldrich) and were used to infect HCC cells. For TIP60 or NR2F6 deletion, GFP^+^ cells were isolated by FACS, and the knockout efficiency was verified by western blotting. For circRHOT1 and NR2F6 promoter deletion, two sgRNAs flanking the target sequence were used at the same time. Next, GFP^+^ cells were isolated by FACS, followed by monoclonalization. Their deletion was verified by DNA sequencing. The sgRNA sequences were listed in Additional file 1: Table S2.

### Antibodies

Anti-GAPDH (5174), anti-Cyclin D1 (2978) anti-TIP60 (12058), anti-P21 (2947), and anti-PCNA (13110) were from Cell Signaling Technology. Anti-Flag (F1804) was purchased from Sigma-Aldrich. Anti-NR2F6 (ab137496) was obtained from Abcam.

### Xenograft assays

For in vivo tumor growth, 1 × 10^6^ HCC cells were subcutaneously injected into the flanks of 5-week-old nude mice from HFK Biosciences (*n* = 6 for each group). The tumor volumes and weights were measured at the indicated time points. Animal assays were approved by the ethnic committee of the Affiliated Hospital of Guilin Medical University.

### Cell proliferation assay

Cell proliferation was measured using CCK-8 assays as previously reported [16]. Briefly, 1 × 10^3^ cells were seeded in 96-well plates and were cultured for 24 h, 48 h, 72 h and 96 h, followed by incubation with 10 μl of CCK-8 assay solution in each well for 2 h. The absorbance values at 450 nm were then measured using an enzyme immunoassay analyzer (Thermo Fisher Scientific, Inc., Waltham, MA, USA). This assay was repeated three times.

### Transwell assays

Cell migration and invasion assays have been described previously [17]. Briefly, 2 × 10^3^ HCC cells were planted in the upper chamber (Mitrogel (BD) pre-coated for invasion) with 200 μl serum-free medium. The lower chamber was added with complete medium. Forty-eight hours later, cells in the lower chamber were fixed and stained with crystal violet (0.2%). Cell numbers were determined using a microscope. This assay was repeated three times.

### Real-time quantitative PCR

TRIzol reagent was used to extract RNA from HCC tissues or cell lines according to the manufacturer’s protocol. Then 1 μg RNA was used to synthesize cDNA, followed by analysis of gene expression on ABI 7300 qPCR system. Relative expression was normalized to *ACTB* or U6. The analysis of circRHOT1 was carried out using convergent primers (Forward, 5′-ATCACCATTCCAGCTGATGT-3′ and reverse, 5′-TGCTGTCTTTGTCTGTTCTTTC-3′) and divergent primers (Forward, 5′-AGTCGATGGATTCCTCTCAT-3′ and Reverse, 5′-AAGTTGTTCATCACTCTGTT-3′). The sequences of other primer are listed in Additional file 1: Table S3.

### Chromatin immunoprecipitation (ChIP) assay

ChIP assays were conducted as previously described [15]. Briefly, HCC cells were fixed with 1% formaldehyde at 37 °C for 10 min and lysed. The genome was sonicated to ~ 500 bp fragments. Anti-TIP60 were added into the lysates and incubated overnight at 4 °C, followed by incubation with Protein A Agarose/Salmon Sperm DNA (50% Slurry) beads for 4 h. Finally, precipitants were eluted and analyzed by qPCR. Primer sequences were listed in Additional file 1: Table S4.

### Electrophoretic mobility shift assay (EMSA)

For RNA EMSAs, the biotin-labeled circRHOT1 RNA probe was obtained by in vitro transcription using the Biotin RNA Labeling Mix (Roche). The LightShift Chemiluminescent RNA EMSA Kit (Thermo Scientific) was used for the shift assay according to the manufacturers’ instructions. This assay was repeated three times.

### RNA in situ hybridization

CircRHOT1 expression in HCC tissues was measured using biotin-labeled circRHOT1 probes. Paraffinized sections were deparaffinized with xylene and 100% ethanol. The sections were then incubated with biotin-labeled probes for 18 h at 40 °C. The DAB substrate was used for the colorimetric detection of circRHOT1. Finally, the sections were co-stained with hematoxylin, followed by dehydration in graded alcohols and xylene. Biotin-conjugated probes were purchased from Invitrogen. The CircRHOT1 probe sequence was as follow: 5′-GGAGGAACCTGCTGTCTTTGTCTGTTC-3′. This assay was repeated three times.

### Fluorescence in situ hybridization

Hybridization was performed overnight with circRHOT1 probes. Specimens were analyzed using a Nikon inverted fluorescence microscope. The circRHOT1 probe for fluorescence in situ hybridization (FISH) is 5′-GGAGGAACCTGCTGTCTTTG-3′. This assay was repeated three times.

### Northern blotting

RNAs were isolated from HCC samples using TRIZOL (Invitrogen). CircRHOT1, linear RHOT1 and 18S probes for northern blotting were achieved using the Biotin RNA labeling mix (Roche). The RNA samples were separated by electrophoresis and were transferred to NC membranes, which were then incubated with the hydration buffer containing the probes. Finally, the RNA signal was detected using the Chemiluminescent Nucleic Acid Detection Module (Thermo Scientific). This assay was repeated three times.

### Pulldown and mass spectrometry

RNA pulldown and mass spectrometry were performed as described before [15]. This assay was repeated three times.

### Minigene assay

This assay was performed according to a previous study [18]. In brief, the sequence of circRHOT1 flanked by two complementary regions (left and right) or only by the left complementary region was inserted into overexpressing vector. Then plasmids were transfected into circRHOT1-depleted 293 T cells and the level of circRHOT1 was measured by northern blotting.

### Analysis of online datasets

The normalized circRNA expression values in two online-available datasets (GSE94508 and GSE97332) were downloaded from GEO database. Then we first searched differentially expressed circRNAs based on the dataset GSE94508. We selected upregulated circRNAs in tumor tissues according to the dataset GSE94508. Then, we analyzed whether these selected circRNAs were still upregulated in the dataset of GSE97332. Finally, circRNAs that were upregulated in these two datasets were determined. Thus, the most differentially expressed circRNAs between HCC tissues and normal tissues were screened out. Afterwards, we analyzed whether these circRNAs were conserved between human and mouse according to Yang’s cohort, which is directly available in their published article [19]. So circRHOT1 was eventually selected. The heatmaps were determined according to the normalized expression levels of circRNAs.

### RNA sequencing

WT and circRHOT1-deficient HCC cells were lysed with TRIZOL reagent, and total RNA was extracted according to the standard method. Next, RNA sequencing was performed (Shenzhen, BGI Genomics Co., Ltd.), and target genes were analyzed.

### Statistical analysis

All results from at least three independent experiments were analyzed using SPSS software version 20.0 (SPSS Inc., Chicago, IL, USA) or GraphPad Prism 6, and expressed as means ± SD. One-way analyses of variance, chi-squared tests, and two-tailed Student’s t-tests were utilized to calculate statistical significance, as appropriate. Survival rate was determined using Kaplan–Meier method and analyzed with the log-rank test. *P* < 0.05 was considered statistically significant.

## Results

### CircRHOT1 is overexpressed in HCC tissues

**Fig. 1:**
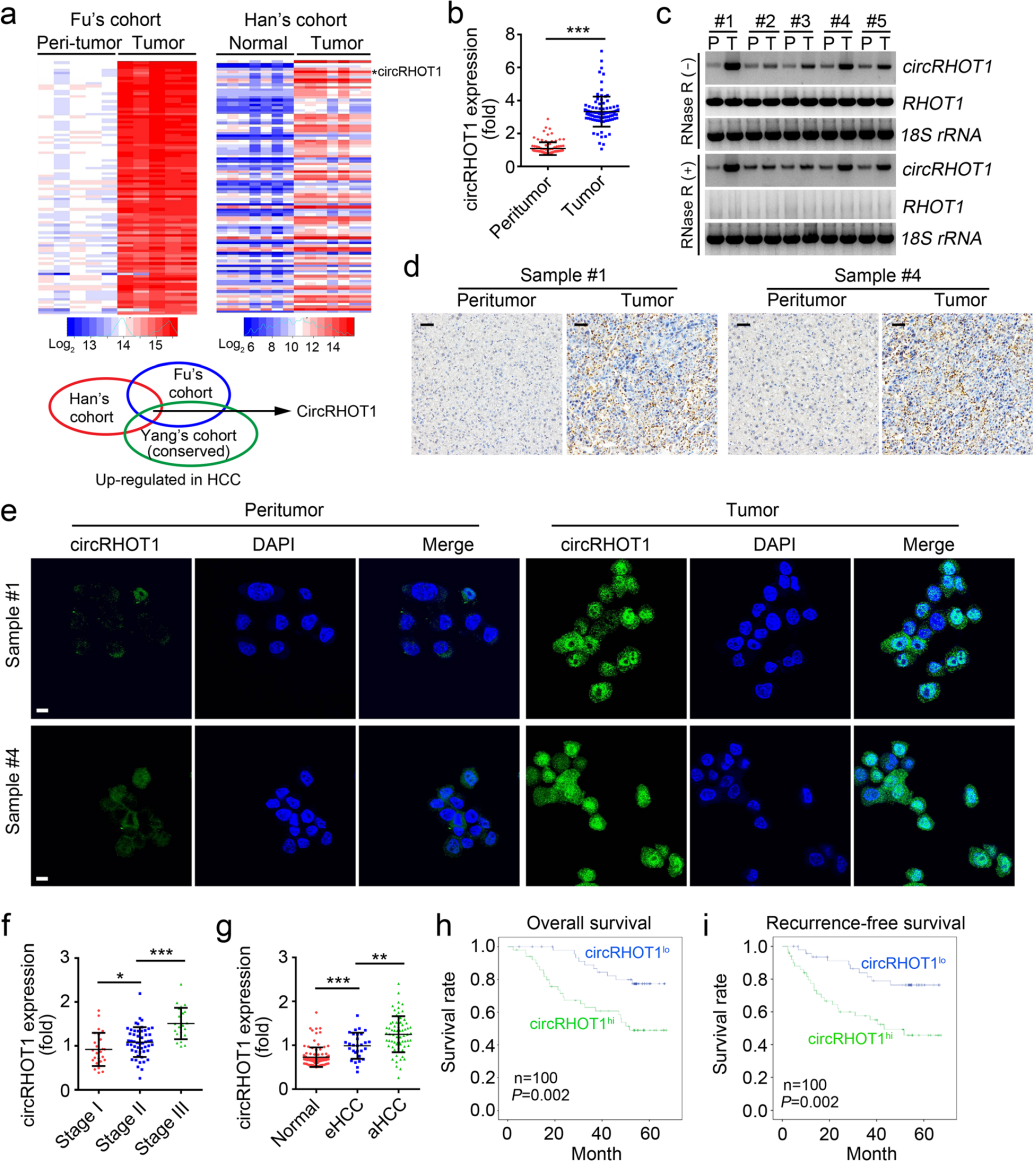
circRHOT1 is overexpressed in HCC tissues. **a** Heatmap of upregulated circRNAs in HCC tissues according to Han’s and Fu’s cohorts. circRHOT was both significantly upregulated and conserved in the two cohorts. **b** The relative expression levels of circRHOT in tumor tissues and adjacent peri-tumor tissues from 100 pairs of HCC patient samples. **c** circRHOT expression was analyzed by northern blotting using 5 paired HCC samples. P, peri-tumor; T, tumor. circRHOT1 was detected using backsplice junction probe. *RHOT1* mRNA was detected using *RHOT1* specific probe. 18S was a loading control and RNA samples for 18S detection were not treated with RNase R. **d**, **e** The expression of circRHOT was determined by in situ hybridization (ISH) (**d**) and RNA fluorescence in situ hybridization (RNA FISH) (**e**) using HCC samples #1 and #4. The scale bars for ISH were 50 μm. The scale bars for FISH were 10 μm. **f** The expression of circRHOT was examined in different clinical stages of HCC samples by qRT-PCR. **g** The relative expression levels of circRHOT in the peritumor, early HCC (eHCC) and advanced HCC (aHCC) were determined by qRT-PCR. **h**, **i** The 100 HCC samples were divided into high and low groups based on circRHOT expression. Next, Kaplan–Meier survival analyses were conducted to determine the overall survival rate (**h**) and recurrence-free survival rate (**i**). *p < 0.05, **p < 0.01 and ***p < 0.001. All the data are representative of at least three independent experiments and presented as the means ± SD

To identify essential circRNAs expressed in HCC tissues, we analyzed online-available datasets concerning the circRNA profiles in HCC tissues (GSE94508 and GSE97332) [7, 20]. We found that many circRNAs are overexpressed in HCC tissues compared to peritumor tissues (Fig. 1a). Among them, circRHOT1 is one of the most upregulated and conserved circRNAs according to Yang’s cohort [19]. Firstly, we validated its features as a circRNA by PCR (Additional file 1: Figure S1a) and RNA sequencing (Additional file 1: Figure S1b). Then, the levels of circRHOT1 in 100 paired HCC tissues and peritumor tissues were determined. Results confirmed that circRHOT1 was significantly upregulated in tumor tissues (Fig. 1b). Next, we chose 5 pairs of HCC sample tissues to subject to northern blotting and validated circRHOT1 was markedly overexpressed in tumor tissue (Fig. 1c). Notably, the linear RHOT1 mRNA level was only slightly elevated as determined by online available datasets and qRT-PCR analysis (Additional file 1: Figure S1c). However, the change on RHOT1 protein level was not obvious in HCC tissues (Additional file 1: Figure S1d). In situ hybridization (ISH) and fluorescence in situ hybridization (FISH) using a biotin-labeled specific probe were further carried out to measure circRHOT1 expression. Results indicated that more circRHOT1 was present in HCC tissues (Fig. 1d, e). In addition, circRHOT1 level was increased in HCC cell lines (Additional file 1: Figure S1e). To investigate the relationship between circRHOT1 level and clinical severity, we checked circRHOT1 expression in HCC tissues with different stages by qRT-PCR. As shown in Fig. 1f, circRHOT1 was expressed more highly in stage III tissues than in stage I/II samples. Moreover, the expression of circRHOT1 in advanced HCC (aHCC) tissues was higher than that in early HCC (eHCC) tissues (Fig. 1g). Next, we investigated the correlation of circRHOT1 expression with the prognosis of HCC patients. We found that HCC patients with higher circRHOT1 expression showed a poorer prognosis by Kaplan–Meier survival analysis (Fig. 1h, i). Taken together, our data suggest that circRHOT1 is highly expressed in HCC tissues and correlated with a poor prognosis.

### CircRHOT1 knockout suppresses HCC cell proliferation, migration and invasion but promotes apoptosis

**Fig. 2:**
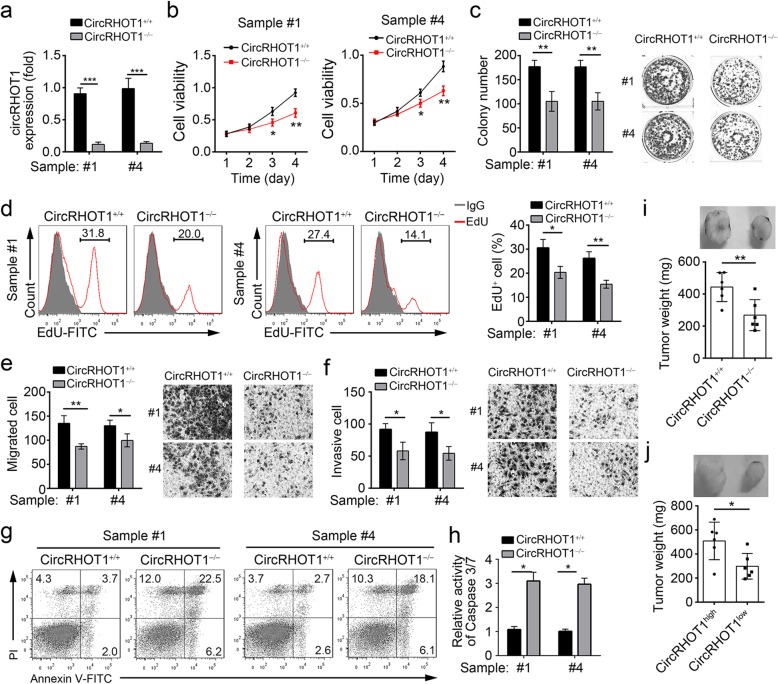
circRHOT1 knockout suppresses HCC cell proliferation, migration and invasion but promotes apoptosis. **a** The expression of circRHOT1 was analyzed by qRT-PCR in circRHOT1-knockout HCC cells. **b**-**d** The effect of circRHOT1 on HCC cell proliferation was determined by CCK-8 (**b**), colony formation (**c**) and EdU incorporation assay (**d**). **e**, **f** The effect of circRHOT1 on invasion (e) and migration (f) was evaluated by transwell assays. **g** The apoptosis of HCC cells was measured by staining with Annexin V/PI, followed by FACS analysis. **h** The Caspase 3/7 activity was measured by using a Caspase 3/7 Activity Apoptosis Assay Kit. **i** WT and circRHOT1-deleted HCC cells were subcutaneously injected into nude recipient mice. Four weeks later, the tumor weights were measured. **j** circRHOT1 low- and high-expression HCC cells were subcutaneously injected into nude recipient mice. Four weeks later, the tumor weights were measured. *p < 0.05, **p < 0.01 and ***p < 0.001. All the data are representative of at least three independent experiments and presented as the means ± SD

We next explored the role of circRHOT1 in HCC progression. A previous study showed that the flanked complementary elements are essential for the formation of circular RNAs [21]. We identified three complementary elements flanking circRHOT1 (Additional file 1: Figure S2a), and only the deletion of the complementary region #3 in the right flank of circRHOT1 exon 6 abrogated the expression of circRHOT1 as determined by minigene assay (Additional file 1: Figure S2a). Thus, we constructed circRHOT1-deficent HCC cell lines through deleting the region #3 in the right flank of circRHOT1 by CRISPR/Cas9 technology. We confirmed the efficiency of circRHOT1 deletion by PCR (Additional file 1: Figure S2b), Northern blotting (Additional file 1: Figure S2c) and qRT-PCR (Fig. 2a). Notably, circRHOT1 deletion did not affect the expression of RHOT1 mRNA and protein (Additional file 1: Figure S2c, d). Then, we performed CCK-8, colony formation and EdU incorporation assays to assess the effect of circRHOT1 on cell proliferation. As shown, circRHOT1 knockout significantly inhibited cell proliferation, colony formation and EdU incorporation (Fig. 2b-d). Next, we conducted Transwell assays and found that circRHOT1 knockout significantly suppressed the migration and invasion of HCC cells (Fig. 2e, f). Moreover, circRHOT1 deletion dramatically promoted the apoptotic cell percentage (Fig. 2g), which is further confirmed by the detection of Caspase 3/7 Activity (Fig. 2h). To further validate the effects of circRHOT1 on HCC cells, we silenced its expression in HCC cell lines, including Hep3B and Huh7 cells (Additional file 1: Figure S2e). Functional experiments from CCK8, colony formation, EdU incorporation, Transwell and FACS results also demonstrated that circRHOT1 knockdown suppressed proliferation, migration and invasion but promoted apoptosis in HCC cells (Additional file 1: Figure S2f-k). Additionally, we conducted rescue assay by re-expressing circRHOT1 in circRHOT1-deleted HCC cells (Additional file 1: Figure S2l). A series of experiments indicated that restoration of circRHOT1 rescued the effects of circRHOT1 knockout on HCC cell proliferation, migration, invasion and survival (Additional file 1: Figure S2m-q), indicating the essential role of circRHOT1 in HCC.

To evaluate the function of circRHOT1 in vivo, we performed xenograft experiments with WT and circRHOT1-deleted HCC cells. As shown in Fig. 2i, circRHOT1 knockout significantly reduced the tumor weight. Furthermore, xenograft experiments with circRHOT1 low- or high-expressed HCC cells showed that circRHOT1 overexpression promoted tumor growth in vivo (Fig. 2j). Collectively, the above data convincingly demonstrated that circRHOT1 promotes HCC development and progression.

### CircRHOT1 regulates NR2F6 expression in HCC cells

**Fig. 3:**
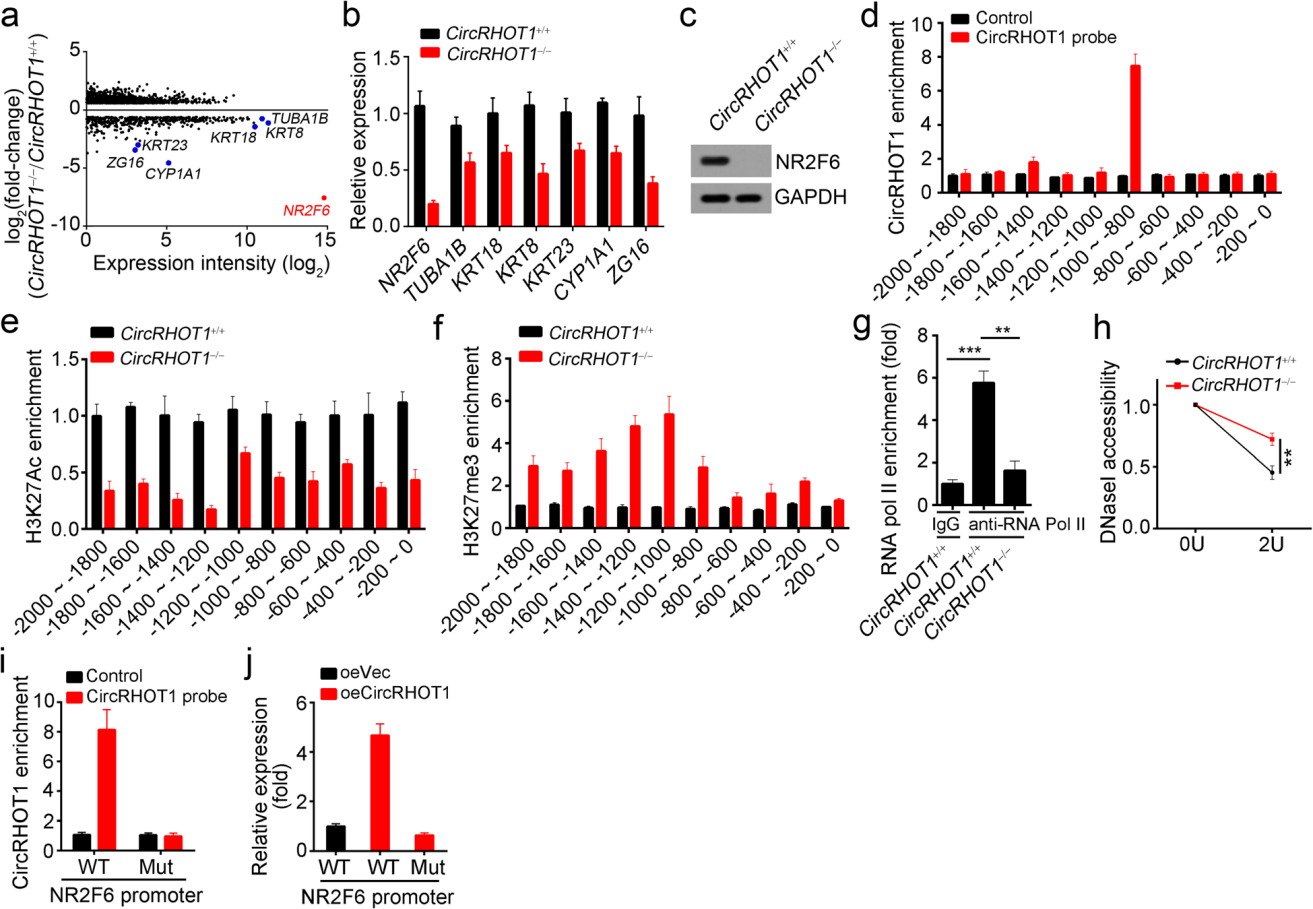
circRHOT1 regulates NR2F6 expression in HCC cells. **a** Bland-Altman plot for differently expressed genes (log_2_(Fold change) > 1.5) and seven selected genes were listed. Red plot denotes NR2F6. **b** The expression of the most downregulated genes was detected by qRT-PCR in WT and circRHOT1-deleted HCC cells. **c** The expression of NR2F6 was determined by western blotting. **d** The enrichment of circRHOT1 on the NR2F6 promoter was assessed by chromatin isolation by RNA purification (CHIRP) using specific biotin-labeled probes (5′-AACCTGCTGTCTTTGTCTGT-3′, 5′-GGAGGAACCTGCTGTCTTTG-3′ and 5′-CCCGGGGAGGAACCTGCTGT-3′) in HCC cells. Control probe sequence is 5′-ATTGTCCGATCGTCTCACGT-3′. **e**-**g** The enrichment of H3K27Ac (**e**), H3K27me3 (**f**) and RNA pol II (**g**) on the NR2F6 promoter was determined by chromatin immunoprecipitation (ChIP) in WT and circRHOT1-deleted HCC cells. **h** The promoter accessibility was evaluated by DNAse I digestion. **i** The binding region of circRHOT1 in the NR2F6 promoter was deleted, and then the enrichment of circRHOT1 was assessed by CHIRP. **j** The mRNA levels of NR2F6 were determined by qRT-PCR in the indicated HCC cells. **p < 0.01 and ***p < 0.001. All the data are representative of at least three independent experiments and presented as the means ± SD

To further investigate the underlying molecular mechanism, we analyzed the differentially expressed genes after circRHOT1 deletion using RNA sequencing. Many genes were downregulated, among which NR2F6 is the most downregulated and possesses high expression value in HCC cells (Fig. 3a). We chose seven downregulated genes based on fold-change and expression level in WT HCC cells to verify the RNA sequencing results by qRT-PCR (Fig. 3b). Notably, these seven genes were also downregulated in Hep3B and Huh7 cells after circRHOT1 silence (Additional file 1: Figure S3a). Western blotting indicated NR2F6 protein level was significantly decreased in circRHOT1-deleted HCC cells (Fig. 3c) and circRHOT1-silenced HCC cell lines (Additional file 1: Figure S3b). Our above data showed that circRHOT1 is mainly distributed in the nucleus (Fig. 1d, e). To determine whether circRHOT1 regulates NR2F6 transcription, we performed chromatin isolation by RNA purification (CHIRP) with specific biotin-labeled circRHOT1 probe and found that circRHOT1 could be enriched on the region of − 1000~ − 800 bp from the transcription start site (TSS) of NR2F6 promoter (Fig. 3d). Moreover, the enrichment of the histone active modification H3K27Ac was reduced after circRHOT1 knockout while that of H3K27me3 was increased (Fig. 3e, f). Consistently, RNA pol II could not bind to the NR2F6 promoter after circRHOT1 deletion (Fig. 3g). Moreover, the promoter of NR2F6 in circRHOT1-deleted HCC cells was more resistant to DNaseI digestion (Fig. 3h), suggesting that circRHOT1 is indispensable for accessibility of the NR2F6 promoter. To further demonstrate the role of the circRHOT1 binding region on NR2F6 promoter, we deleted this region in HCC cells. CircRHOT1 could not enrich on the NR2F6 promoter after this region deletion (Fig. 3i). Additionally, we found that the overexpression of circRHOT1 promoted the mRNA level of NR2F6, while deletion of the binding region abrogated this effect (Fig. 3j). Thus, circRHOT1 was associated with the NR2F6 promoter and promoted its expression.

### CircRHOT1 is associated with TIP60

**Fig. 4:**
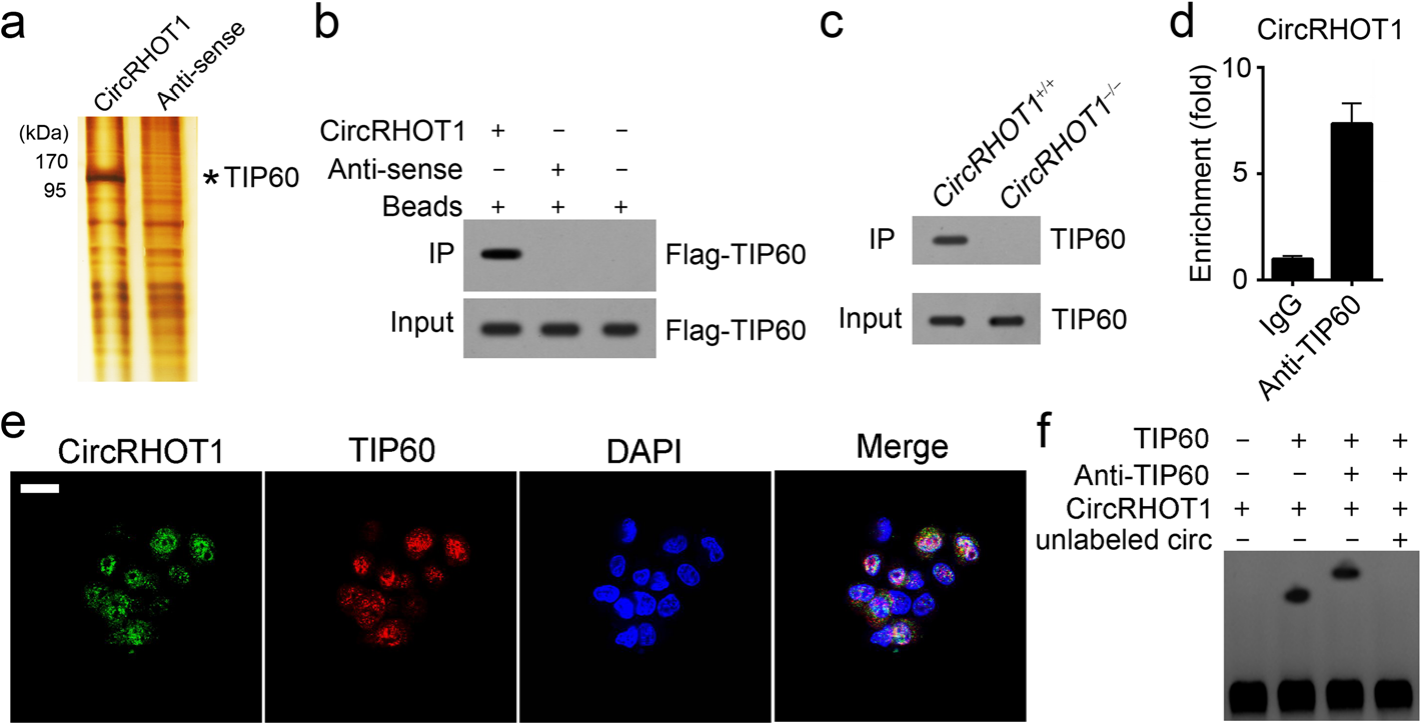
circRHOT1 associates with TIP60. **a** A specific biotin-labeled circRHOT1 probe was added to HCC lysates, and the precipitated proteins were resolved by SDS-PAGE, followed by silver staining. Next, the differential band in the circRHOT1 probe lane was identified by mass spectrometry (MS). The anti-sense of circRHOT1 serves as control. As shown, TIP60 was identified. **b** RNA pulldown was performed to verify the interaction between TIP60 and circRHOT1 using TIP60-overexpressing HCC cells. Biotin-labeled circRHOT1 or anti-sense was used to precipitate Flag-TIP60. **c** The specific biotin-labeled circRHOT1 probe (5′-TCTTCTGCCCGGGGAGGAACCTGCTGTCTTTGTCTGTTCTTTCAT-3′) did not precipitate TIP60 in circRHOT1-knockout HCC cells. **d** RNA IP results indicated that TIP60 enriched circRHOT1 in HCC cells. **e** CircRHOT1 co-localized with TIP60 in HCC cells as shown by RNA fluorescence in situ hybridization (FISH). Scale bar, 10 μm. **f** RNA EMSA assay indicated that TIP60 interacts with circRHOT1 directly. Linear circRHOT1 was labeled with biotin. All the data are representative of at least three independent experiments and presented as the means ± SD

To investigate whether circRHOT1 cooperated with specific proteins to regulate NR2F6 expression, we performed RNA pulldown using HCC cell lysates, followed by silver staining and mass spectrum identification. We identified TIP60 as a potential interactive protein of circRHOT1 (Fig. 4a). Furthermore, RNA pulldown with TIP60-overexpressing HCC cells showed that biotin-labeled circRHOT1 precipitated Flag-TIP60 (Fig. 4b). Moreover, biotin-labeled circRHOT1 probe could precipitate endogenous TIP60 in WT HCC cell lysates (Fig. 4c). Besides, TIP60-specific antibodies enriched endogenous circRHOT1 in HCC cells (Fig. 4d). According to FISH assay, circRHOT1 was co-localized with TIP60 in HCC cells (Fig. 4e). RNA-EMSA assay also demonstrated that TIP60 directly interacts with circRHOT1 (Fig. 4f).

### CircRHOT1 recruits TIP60 to initiate NR2F6 expression

**Fig. 5:**
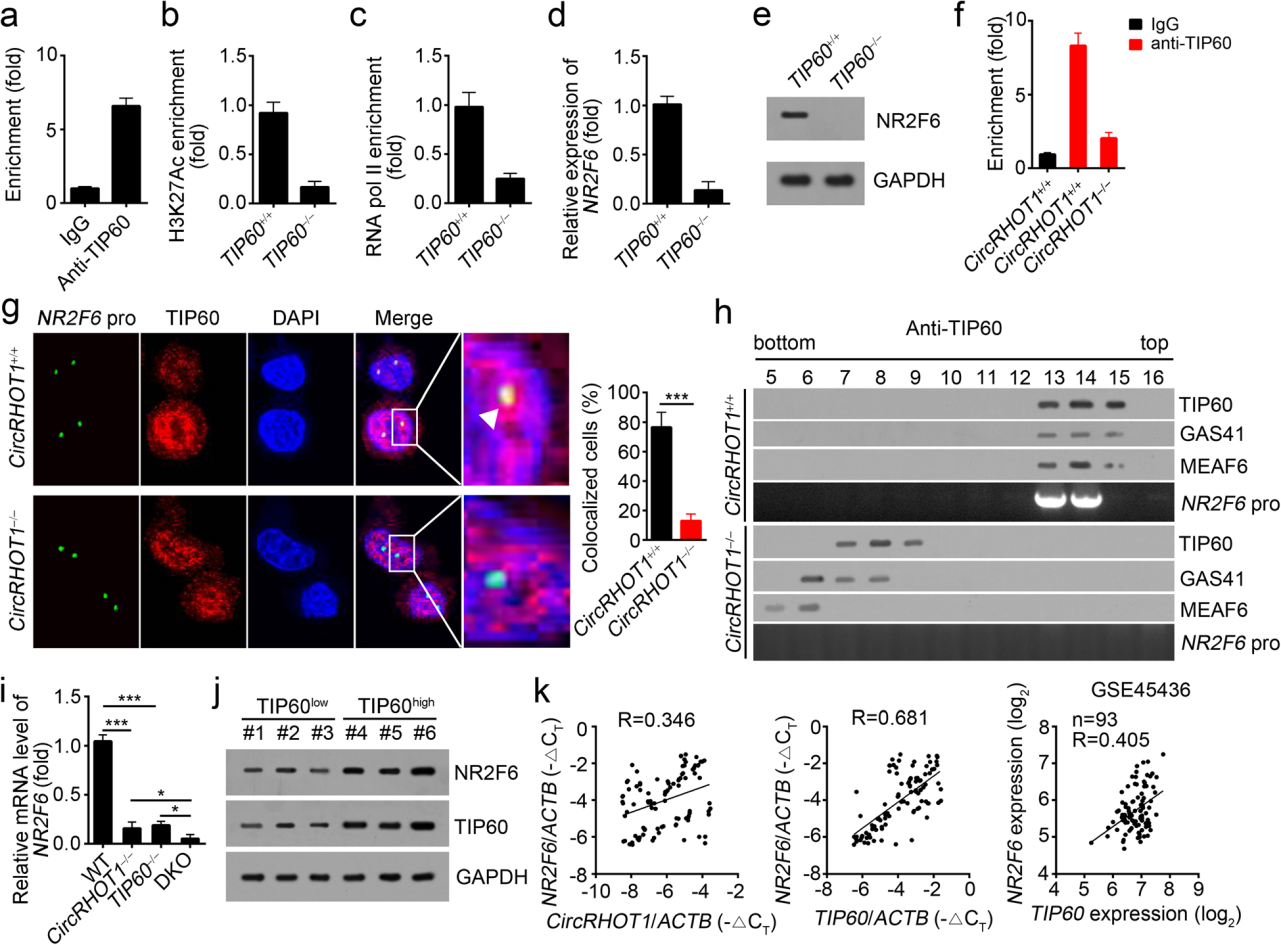
circRHOT1 recruits TIP60 to initiate NR2F6 expression. **a** ChIP assays indicated that TIP60 is associated with the NR2F6 promoter on the same region as circRHOT1. **b**, **c** TIP60 knockout significantly reduced the enrichment of H2K27Ac (**b**) and RNA pol II (**c**) on the NR2F6 promoter. **d**, **e** TIP60 knockout significantly inhibited the mRNA (**d**) and protein levels (**e**) of NR2F6 in HCC cells. **f**, **g** RNA IP (**f**) and RNA FISH (**g**) results showed that circRHOT1 knockout suppresses the association of TIP60 with the NR2F6 promoter in HCC cells. Scale bar, 10 μm. **h** The indicated HCC cells were lysed and treated with 1% formaldehyde for crosslinking. Next, anti-TIP60 was incubated with the treated lysates for ChIP assays, followed by size fractionation via sucrose gradient ultracentrifugation. The eluate gradients were examined by western blotting and PCR assays. Pro: promoter. **i** circRHOT1 and TIP60 double knockout suppressed the mRNA level of NR2F6 more seriously. **j** The protein levels of TIP60 and NR2F6 were determined by western blotting in HCC tissues. **k** The expression of NR2F6 was inversely correlated with the expression of circRHOT1 and TIP60 in HCC tissues by qRT-PCR and according to the GSE45436 dataset. ***p < 0.001. All the data are representative of at least three independent experiments and presented as the means ± SD

To further determine whether TIP60 participates in the regulation of NR2F6 expression, we performed ChIP assays with HCC cells. We found that TIP60 was enriched in the same region of the NR2F6 promoter as circRHOT1 (Fig. 5a). Furthermore, TIP60 knockout also inhibited the enrichment of H3K27Ac and RNA pol II on the NR2F6 promoter (Fig. 5b, c). In addition, TIP60 deletion downregulated the mRNA and protein levels of NR2F6 in HCC cells (Fig. 5d, e). Next, we wanted to determine the role of circRHOT1 in TIP60-mediated expression of NR2F6. We found that circRHOT1 knockout abrogated the enrichment of TIP60 on the promoter of NR2F6 (Fig. 5f). DNA FISH also indicated that circRHOT1 knockout abolished the co-localization of TIP60 with the NR2F6 promoter (Fig. 5g). Importantly, circRHOT1 deficiency lost the recruitment of the NuA4 complex to the *NR2F6* promoter, whereas the NuA4 complex bound to the *NR2F6* promoter in control HCC cells (Fig. 5h). Moreover, double knockout of circRHOT1 and TIP60 further inhibited the mRNA level of NR2F6 in HCC cells (Fig. 5i). We then analyzed the relationships among the expression levels of circRHOT1, TIP60 and NR2F6 in HCC tissues. We found that the protein and mRNA levels of NR2F6 were positively correlated with those of TIP60 in HCC tissues by qRT-PCR and according to the GSE45436 dataset (Fig. 5j, k). Additionally, there was a positive correlation between the expression of NR2F6 and circRHOT1 in HCC tissues (Fig. 5k and Additional file 1: Figure S3c) while no significant expression correlation between circRHOT1 and TIP60 existed (Additional file 1: Figure S3c). Altogether, circRHOT1 is essential for TIP60-mediated NR2F6 expression in HCC tissues.

### Restoration of NR2F6 rescues circRHOT1 deletion-mediated suppression of HCC growth and metastasis

**Fig. 6:**
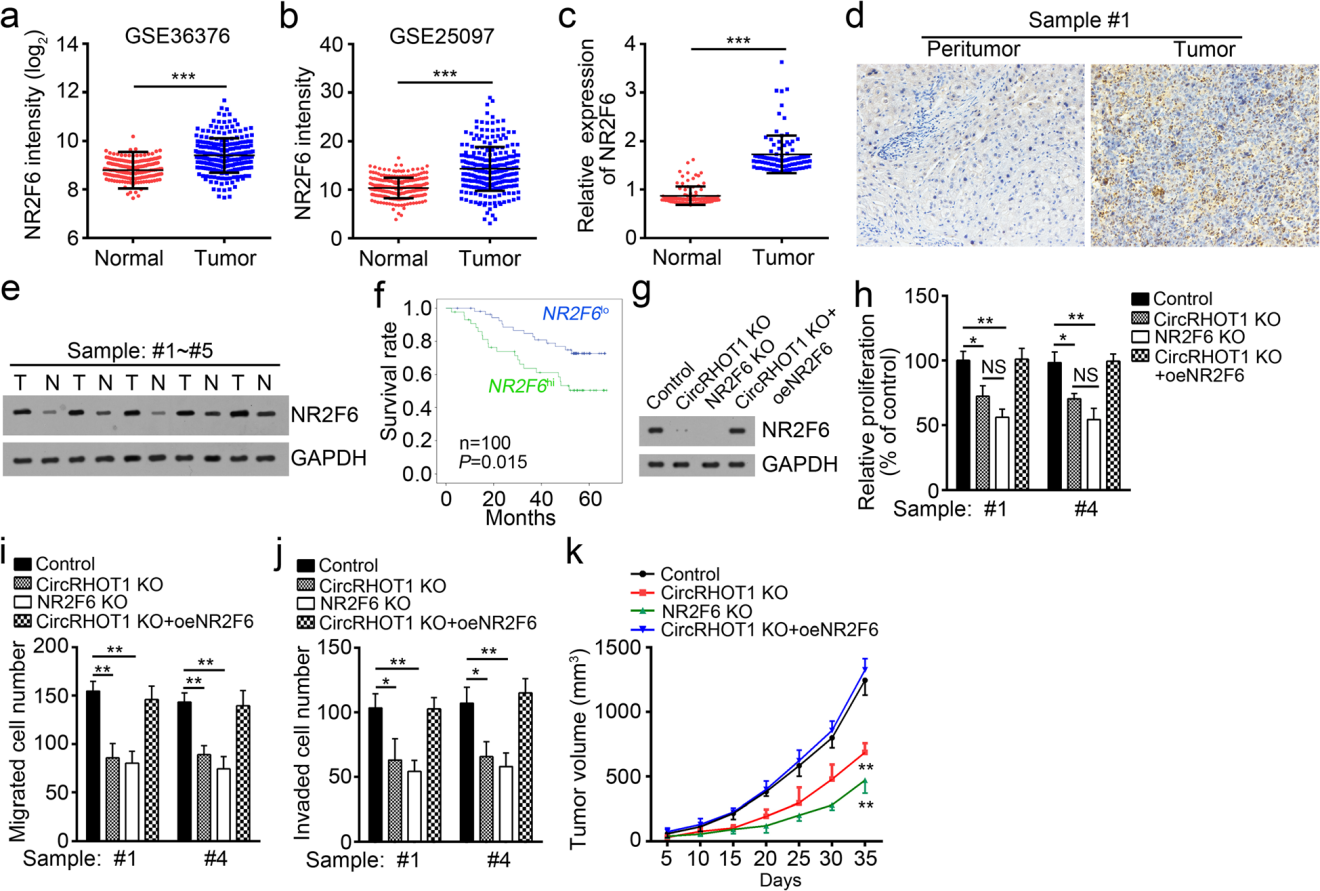
Restoration of NR2F6 rescues circRHOT1 deletion-mediated suppression of HCC growth and metastasis. **a**-**c** NR2F6 mRNA levels were upregulated in HCC tissues compared with those in adjacent normal tissues according to GSE36376 dataset (**a**), GSE25097 dataset (**b**) and qRT-PCR results (**c**). **d**, **e** Immunohistochemical analysis (**d**) and western blotting (**e**) indicated that the NR2F6 protein level is upregulated in HCC tissues. **f** Kaplan–Meier survival analyses were conducted to assess the prognostic significance of NR2F6 expression in HCC patients. **g** The NR2F6 protein level was restored by transduction with NR2F6 ectopically expressing plasmid in circRHOT1-knockout HCC cells. **h**-**j** The effects of NR2F6 on HCC cell proliferation (**h**), migration (**i**) and invasion (**j**) were assessed by CCK-8 and Transwell assays. **k** The effect of NR2F6 on tumor growth in vivo was determined by xenograft assays. *p < 0.05, **p < 0.01 and ***p < 0.001. All the data are representative of at least three independent experiments and presented as the means ± SD

To further explore the role of NR2F6, we examined its expression in HCC tissues. According to Park’s cohort (GSE36376) [22] and Zhang’s cohort (GSE25097) [23–25], NR2F6 was upregulated in HCC tissues compared with that in adjacent normal tissues (Fig. 6a, b). Furthermore, we examined the expression of NR2F6 in 100 paired HCC samples and found that NR2F6 was upregulated in tumor tissues (Fig. 6c). Immunohistochemical analysis and western blotting confirmed that NR2F6 level was elevated in HCC tissues (Fig. 6d, e). Moreover, higher expression of NR2F6 in HCC patients suggested a poorer prognosis (Fig. 6f). We then knocked out NR2F6 or restored its protein level in circRHOT1-deleted HCC cells (Fig. 6g). CCK-8 and Transwell assays revealed that knockout of either circRHOT1 or NR2F6 inhibited cell proliferation, migration and invasion, while restoration of NR2F6 in circRHOT1-deleted HCC cells rescued cell proliferation, migration and invasion in vitro (Fig. 6h-j). In vivo xenograft experiments showed that knockout of circRHOT1 or NR2F6 suppressed tumor growth. However, restoration of NR2F6 rescued circRHOT1-deletion-mediated inhibition on tumor growth (Fig. 6k). Thus, our results demonstrated that circRHOT1 inhibits HCC progression via the activation of NR2F6.

## Discussion

With the advancement on high-throughput sequencing technology, increasing circRNAs have been identified in human tissues [26]. The study concerning the functions of circRNAs has attracted increasing attention. Many reports have revealed a close correlation between circRNA expression and human diseases, especially in tumorigenesis [27, 28]. However, the functions of circRNAs in HCC remain elusive. In this study, we screened out an uncharacteristic and upregulated circRNA named circRHOT1 in HCC using a bioinformatics method. Additionally, we investigated the function and mechanism of circRHOT1 during HCC development.

Currently, many molecules, including proteins and non-coding RNAs, have been identified as potential biomarkers for HCC diagnosis or prognosis [29]. However, whether circular RNAs could be good biomarkers remains to be further investigated. In our study, we showed that circRHOT1 expression was upregulated in HCC tissues. And increased expression of circRHOT1 in HCC patients predicted a poor prognosis. Therefore, circRHOT1 may serve as a potential biomarker for HCC prognosis. Our results were from 100 HCC patients. Thus, to determine the practical value of circRHOT1 expression for evaluating HCC prognosis still needs a larger sample size and further investigation. Additionally, whether circRHOT1 might be a marker for HCC diagnosis must be explored. A previous study showed that circular RNA is stable and exists in exosomes [30]. Whether circRHOT1 is present in exosomes and whether circulating circRHOT1 is also related to HCC remain to be explored. CircRHOT1 is originated from RHOT1 mRNA. However, we found that RHOT1 mRNA level was slightly upregulated in HCC tissues compared to normal tissues, according to online datasets (GSE36376 and GSE25097) and qRT-PCR analysis. Notably, circRHOT1 level was upregulated in HCC tissues over four times (Fig. 1b). Thus, upregulation of circRHOT1 in HCC tissues might be due to both the aberrant transcription of RHOT mRNA itself and the frequent back-splicing event, which requires further validation.

The molecular mechanisms that regulate cancer development and progression have not been fully elaborated. Additionally, how circRNAs participate in tumorigenesis requires further investigation. Many studies have shown that circRNAs could regulate the expression of oncogenes or tumor suppressors mainly via a circRNA-miRNA-mRNA axis [31, 32]. A few reports have also shown that circRNAs may associate with specific proteins to exert important functions in cancer [33, 34]. The regulatory network of circRNAs with other molecules requires more investigation in the process of tumor development. In our study, we showed that circRHOT1 mainly locates in the nucleus. Therefore, we hypothesized whether circRHOT1 interacts with some proteins to promote HCC progression. By MS identification and RNA pulldown, we verified that circRHOT1 associates with TIP60. TIP60 is the major subunit of the NuA4 chromatin remodeling complex, which regulates histone acylation modification and initiates the expression of target genes [35]. Previous studies indicate that TIP60 promotes mammary tumorigenesis [36], pleural mesothelioma malignance [37] and prostate cancer growth [38]. TIP60 is also involved in DNA repair [39]. Whether TIP60 participates in liver cancer remains unclear. Hence, we further explored the mechanism by which circRHOT1 and TIP60 regulate the expression of specific downstream target genes.

By RNA sequencing, we analyzed the downstream regulated genes by circRHOT1. We identified NR2F6 as the most downregulated gene after circRHOT1 knockout. NR2F6 is a member of the NR2F family, which could dimerize with RXR/NR2B1 or other NRs and recognize various DNA response elements [40]. A previous study has indicated that NR2F6 is a regulator of adaptive immunity^31^. NR2F6 regulates CD4^+^ T-cell effector functions and negatively controls cell development [41, 42]. Recent reports showed that NR2F6 is involved in human cancers such as ovarian cancer and cervical cancer [43, 44]. However, the functional role of NR2F6 has not been investigated in HCC. In this study, we demonstrated that NR2F6 is the target gene of circRHOT1 and TIP60 in HCC. By ChIP and FISH assays, we proved that circRHOT1 recruited TIP60 to the NR2F6 promoter, which leads to further recruitment of other components of NuA4 complex by TIP60 and eventually initiated NR2F6 expression. Moreover, we showed that NR2F6 was overexpressed in HCC tissues. Knocking out NR2F6 significantly inhibited HCC growth and metastasis. We also found that higher expression of NR2F6 in HCC patients predicts lower survival rate, indicating NR2F6 might be a prognostic marker. Additionally, restoration of NR2F6 in circRHOT1-deficient HCC cells rescued cell proliferation, migration and invasion. Thus, our study expanded the role of circRNAs and its functional mechanism in HCC. Notably, we observed that either circRHOT1 knockout or NR2F6 knockout suppressed the activation of NOTCH2 pathway (Additional file 1: Figure S3d), suggesting circRHOT1/TIP60/NR2F6 axis might exert roles through NOTCH2 signaling partially. Even so, the downstream mechanism of NR2F6 in HCC requires more exploration. Due to the importance of NR2F6 in other types of cancers [43, 44], whether circRHOT1/TIP60/NR2F6 axis also plays a role in these cancers needs further investigation.

## Conclusion

In summary, our findings suggested that circRHOT1 is highly expressed in HCC tissues and correlated with HCC patients’ prognosis. Mechanistically, circRHOT1 promotes HCC development and progression via TIP60-dependent NR2F6 expression (Additional file 1: Figure S3e). Our results suggest that circRHOT1 may serve as a potential biomarker for HCC prognosis. Notably, due to the difficulty of inhibiting circRHOT1 in HCC patients, it might be more promising to pharmacologically inhibit NR2F6 as a therapeutic target.

## Additional file

Additional file 1: Additional informations. (DOCX 1045 kb)

## Data Availability

The microarray data used in the study (GSE94508, GSE97332, GSE36376 and GSE25097) are available in a public repository from GEO database. The RNA-sequencing raw data and normalized results were submitted to the SRA database (SRP165820) and GEO database (GSE121468).

## Abbreviations

ceRNA: Competing endogenous RNA
circRNA: Circular RNA
FISH: Fluorescence in situ hybridization
GC: Gastric cancer
IHC: Immunohistochemistry
ISH: In situ hybridization
qRT-PCR: Real-time quantitative polymerase chain reaction
RIP: RNA immunoprecipitation
