# Supplementary material for: Circular RNA circRHOT1 promotes hepatocellular carcinoma progression by initiation of NR2F6 expression
Source: Mol Cancer. 2019 Jul 19;18:119. doi: 10.1186/s12943-019-1046-7 (PMC6639939; doi:10.1186/s12943-019-1046-7)
Supplement: Supplementary file 1 — Additional informations. (DOCX 1045 kb) [file 12943_2019_1046_MOESM1_ESM.docx]

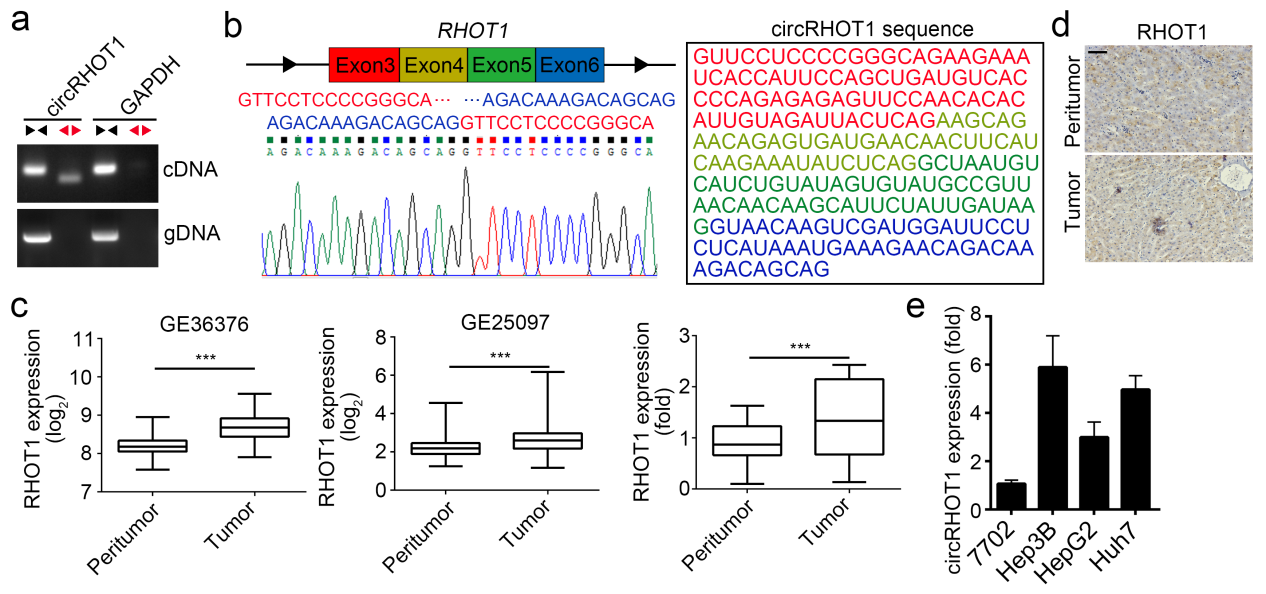


**Figure S1.** circRHOT1 is overexpressed in HCC cell lines. **a** Divergent (red arrowheads) and convergent (black arrowheads) primers were used to amplify circRHOT1 using complementary DNA (cDNA) and genomic DNA (gDNA) as templates. **b** PCR products using divergent primers was sequenced to validate circRHOT1. **c** Expression levels of linear RHOT1 in HCC tissues and peritumor tissues according to online datasets (GSE36376 and GSE25097) and qRT-PCR analysis. **d** RHOT1 protein level was determined using IHC in HCC tissues and paired peritumor tissues. Scale bar, 50 μm. **e** Relative expression of circRHOT1 was examined in HCC cell lines by qRT-PCR. All the data are representative of three independent experiments and are presented as the means ± SD. ****p*<0.001.


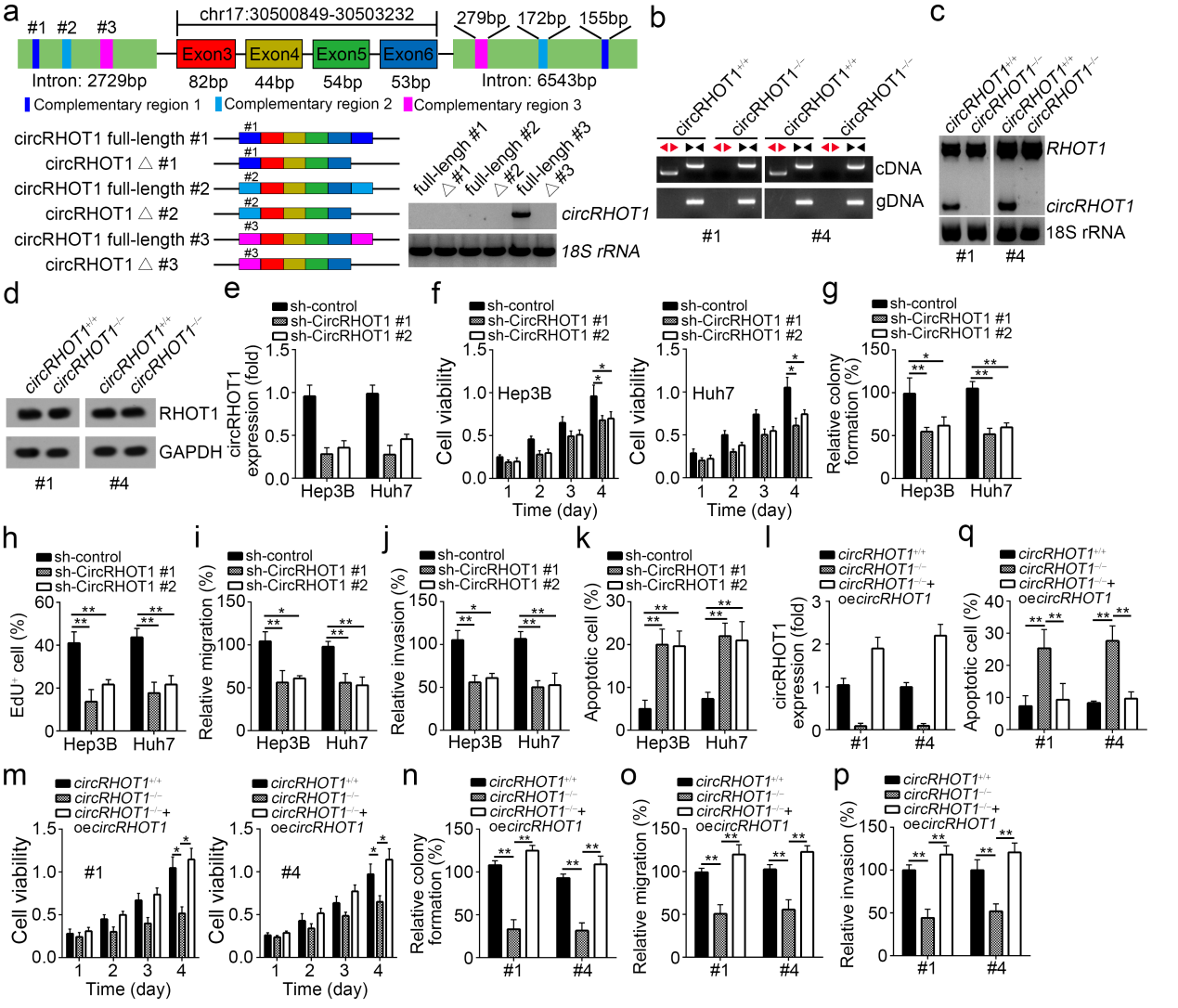


**Figure S2.** circRHOT1 regulates HCC cell malignant behaviors. **a** Diagrammatic sketch of the complementary elements in the flanked introns. CircRHOT1-depleted 293T cells were transfected with indicated minigene constructs and circRHOT1 level was measured by northern blotting. **b** circRHOT1 deletion in HCC cells were validated by PCR. **c** levels of circRHOT1 and RHOT1 mRNA in circRHOT1-deleted HCC cells were measured using Northern blotting. Two different probes targeting circRHOT1 and RHOT1 respectively were used at the same time. **d** protein levels of RHOT1 in circRHOT1-deleted HCC cells were analyzed by western blotting. **e** Relative expression of circRHOT1 in circRHOT1-silenced Hep3B and Huh7 cells by qRT-PCR. (**f-h**) CCK-8 assay (**f**), colony formation assay (**g**) and EdU incorporation assay (**h**) were performed to analyze cellular proliferation after circRHOT1 depletion in Hep3B and Huh7 cells. **i, j** Transwell assay was conducted to determine migration (**i**) and invasion (**j**). **k** Knockdown of circRHOT1 increased the apoptotic cell rate. **l** Relative expression of circRHOT1 in HCC sample cell lines by qRT-PCR. **m, n** Cellular proliferation of HCC sample cell lines were measured by CCK-8 (**m**) and colony formation (**n**) assays. **o, p** Transwell assay was used to analyze cell migration (**o**) and invasion (**p**) in HCC sample cell lines. **q** Re-expression of circRHOT1 reversed increased cell apoptosis caused by circRHOT1 deletion. All the data are representative of three independent experiments and are presented as the means ± SD. **p*<0.05 and **p*<0.01.


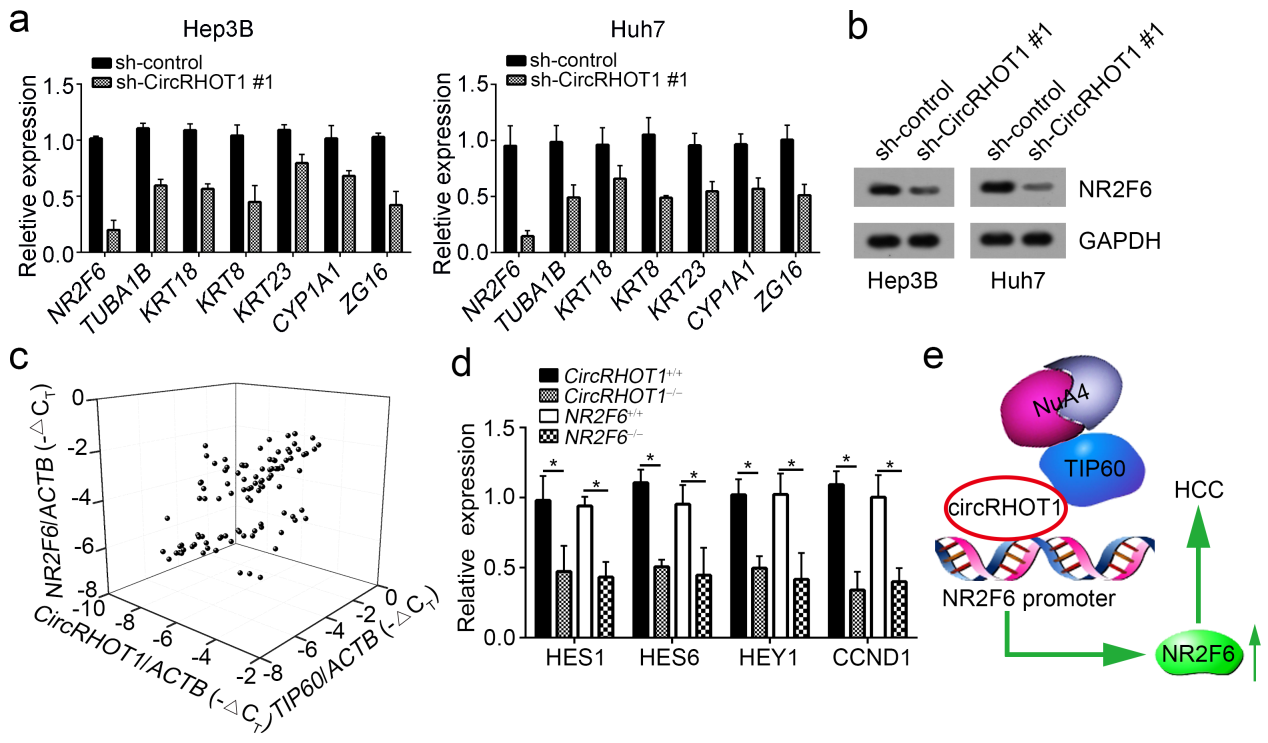


**Figure S3.** circRHOT1 promotes NR2F6 expression. **a, b** qRT-PCR analysis (**a**) and western blotting (**b**) indicates circRHOT1 knockdown suppressed NR2F6 expression in Hep3B and Huh7 cells. **c** 3D correlation between circRHOT1, TIP60 and NR2F6 in HCC tissues. **d** Deletion of either circRHOT1 or NR2F6 suppressed the expression of target genes (HES1, HES6, HEY1 and CCND1) of NOTCH2 pathway in HCC cells. **e** Work model. CircRHOT1 recruits TIP60 to initiate NR2F6 transcription, leading to HCC progression. All the data are representative of three independent experiments and are presented as the means ± SD. **p*<0.05.

**Table S1. Clinical Characteristics and Outcome of 100 HCC Patients According to circRHOT1 Expression Levels**

| Feature | circRHOT1  Low | High* | P value |
| --- | --- | --- | --- |
| All cases | 49 | 51 |  |
| Age |  |  | 0.688 |
| <60 | 27 | 26 |  |
| ≥60 | 21 | 25 |  |
| Gender |  |  | 1.000 |
| Male | 41 | 43 |  |
| Female | 8 | 8 |  |
| AFP |  |  | 0.687 |
| <400 | 23 | 21 |  |
| ≥400 | 26 | 30 |  |
| Size (cm) |  |  | 0.027 |
| <3 | 15 | 6 |  |
| ≥3 | 34 | 45 |  |
| BCLC |  |  | 0.306 |
| A | 22 | 17 |  |
| B or C | 27 | 34 |  |
| TNM |  |  | 0.017 |
| I/II | 43 | 34 |  |
| III/IV | 6 | 17 |  |

*The median expression level was used as the cutoff.

For analysis of correlation between circRHOT1 levels and clinical features, Pearson’s chi-square tests were used. Results were considered statistically significant at *P*<0 .05.

Abbreviations: AFP, alpha-fetoprotein; BCLC, Barcelona Clinic liver cancer staging system; TNM, tumor-node metastasis.

**Table S2. sgRNA sequence for knockout**

| Gene | sequence |
| --- | --- |
| *circRHOT1 #1* | 5’-AGTCGATCGACCACCCAGCT-3’ |
| *circRHOT1 #2:* | 5’-AGCCATCTGCAGGCTCGATG-3’ |
| *TIP60* | 5’-ATGGGTGACGCATGAGCGGC-3’ |
| *NR2F6* | 5’-TCCGGTGGTGCTGGTCGATC-3’ |
| *NR2F6 promoter #1:* | 5’-TCGGGTTAACACCTGGTGCC-3’ |
| *NR2F6 promoter #2* | 5’-GGGCTTGTCCAGCCGGTTAA-3’ |

**Table S3. Real time PCR primers used in this study**

| Genes | Forward | Reverse |
| --- | --- | --- |
| *ACTB* | 5’-TCCATCATGAAGTGTGACGT-3’ | 5’-GAGCAATGATCTTGATCTTCAT-3’ |
| *NR2F6* | 5’-TCCAGGATGGAGGGTCCAAT-3’ | 5’-CCCACCATCCCACAAGTTCA-3’ |
| *TUBA1B* | 5’-AAGCGTGCCTTTGTTCACTG-3’ | 5’-GACATGCTGCAGGGCCAAAA-3’ |
| *KRT8* | 5’-GCGGAATGAATGGGGTGAGC-3’ | 5’-CTGGGTCACCCTGATGGACA-3’ |
| *KRT23* | 5’-AGGCATCAGATATTCACCACCTTCT-3’ | 5’-CCATCCACTATCTCTAAAGGGAGG-3’ |
| *CYP1A1* | 5’-CCAGCTCAGCTCAGTACCTC-3’ | 5’-GCCGACATGGAGATTGGGAA-3’ |
| *KRT18* | 5’-TGCGATATAACTCGGGTCGC-3’ | 5’-GAGCGAGTGGTGAAGCTCAT-3’ |
| *ZG16* | 5’-GGGGACGTGCAAATCTCACT-3’ | 5’-GAAGAGCGTTGATGCAAGCC-3’ |
| *TIP60* | 5’-CACTGGCTGTGCACGTTATG-3’ | 5’-CGGGGGAAAACGGAGTGTTA-3’ |

**Table S4. Primer sequences targeting NR2F6 promoter in ChIP assay**

| Region | Forward | Reverse |
| --- | --- | --- |
| *-2000~-1800* | 5’-AAGACTTGTCTCCCCCAAACCT-3’ | 5’-CGTGAGCAAGACCCTGTCTCA-3’ |
| *-1800~-1600* | 5’-TTATTGCCCCGGCTGGTCTTGA-3’ | 5’-GCTGTGGCTCACGCCTGTAAT-3’ |
| *-1600~-1400* | 5’-ATCCCAAATTACCAGTCACATT-3’ | 5’-GGCGTGGGTGGGCAGAGACGA-3’ |
| *-1400~-1200* | 5’-CGTCCCTGGAGCAGGTTGAGAG-3’ | 5’-AGATTCTTAGAGATCTTACA-3’ |
| *-1200~-1000* | 5’-AGGCACTGTCCTGGCCAGAG-3’ | 5’-GTGGTCCCACCCCGCCCTGA-3’ |
| *-1000~-800* | 5’-TGCTTAATAAATGCTGGTCG-3’ | 5’-CAGATGTCTCTTCCAGGCGC-3’ |
| *-800~-600* | 5’-AGACAGGGCATGCCTGTCGCC-3’ | 5’-CTGGCCGGGGCCGCGAGGTC-3’ |
| *-600~-400* | 5’-CGGCGACTTTCTGGAAGAGGA-3’ | 5’-TACGCCCCTGCGCAAGACA-3’ |
| *-400~-200* | 5’-GAGCGACCCAACGTCCAAGGCCA-3’ | 5’-TTCCTTGAGCCTCGCCAAGAT-3’ |
| *-200~0* | 5’-CCAGCGCTCGGCCCCCGTCC-3’ | 5’-CCGGCGACGGCCGCCCTTATAA-3’ |
